# Extract of *Scutellaria baicalensis* inhibits dengue virus replication

**DOI:** 10.1186/1472-6882-13-91

**Published:** 2013-04-29

**Authors:** Keivan Zandi, Tong-Hye Lim, Nor-Aziyah Rahim, Meng-Hooi Shu, Boon-Teong Teoh, Sing-Sin Sam, Mohammed-Bashar Danlami, Kim-Kee Tan, Sazaly Abubakar

**Affiliations:** 1 Tropical Infectious Disease Research and Education Center (TIDREC), Department of Medical Microbiology, Faculty of Medicine, University of Malaya, Kuala Lumpur, Malaysia; 2 Herbitec Sendirian Berhad, G-3-7, Plaza Damas Jalan Sri Hartamas, Sri Hartamas, Kuala Lumpur, Malaysia

**Keywords:** Infectious disease, Antiviral, Dengue, Flavonoids, Baicalein, *Scultellaria baicalensis*

## Abstract

**Background:**

*Scutellaria baicalensis* (*S. baicalensis*) is one of the traditional Chinese medicinal herbs that have been shown to possess many health benefits. In the present study, we evaluated the *in vitro* antiviral activity of aqueous extract of the roots of *S. baicalensis* against all the four dengue virus (DENV) serotypes.

**Methods:**

Aqueous extract of *S. baicalensis* was prepared by microwave energy steam evaporation method (MEGHE™), and the anti-dengue virus replication activity was evaluated using the foci forming unit reduction assay (FFURA) in Vero cells. Quantitative real-time polymerase chain reaction (qRT-PCR) assay was used to determine the actual dengue virus RNA copy number. The presence of baicalein, a flavonoid known to inhibit dengue virus replication was determined by mass spectrometry.

**Results:**

The IC_50_ values for the *S. baicalensis* extract on Vero cells following DENV adsorption ranged from 86.59 to 95.19 μg/mL for the different DENV serotypes. The IC_50_ values decreased to 56.02 to 77.41 μg/mL when cells were treated with the extract at the time of virus adsorption for the different DENV serotypes. The extract showed potent direct virucidal activity against extracellular infectious virus particles with IC_50_ that ranged from 74.33 to 95.83 μg/mL for all DENV serotypes. Weak prophylactic effects with IC_50_ values that ranged from 269.9 to 369.8 μg/mL were noticed when the cells were pre-treated 2 hours prior to virus inoculation. The concentration of baicalein in the *S. baicalensis* extract was ~1% (1.03 μg/gm dried extract).

**Conclusions:**

Our study demonstrates the *in vitro* anti-dengue virus replication property of *S. baicalensis* against all the four DENV serotypes investigated. The extract reduced DENV infectivity and replication in Vero cells. The extract was rich in baicalein, and could be considered for potential development of anti-DENV therapeutics.

## Background

Many traditionally used herbs contain phytochemicals that could be beneficial for health. These plant-derived compounds and extracts have been used in many forms for the treatment of various ailments and as dietary supplements [1-3]. Traditionally used herbal preparations commonly used by different communities across the world, have been the immediate source of knowledge for further discovery of potentially beneficial phytochemicals. It is a widely believed that naturally-derived phytochemicals would have lesser side-effects as they are often derived from edible herbs [4-6]. The traditional Chinese medicinal practices advocate the use of herbs, many of which have been extensively characterized and studied [7,8]. Among these herbs include those that have been described to possess anti-infective properties [9-11]. Traditionally, most of these herbal-based medications are dispensed in aqueous forms, and be administered orally [12]. Given the availability of huge number of traditionally used medicinal plants, it is likely that at least a few of these would exhibit antiviral activities at concentrations suitable for direct consumption [13].

*S. baicalensis* is one of the most widely used medicinal plants, and is officially listed in the Chinese Pharmacopoeia [14]. Extracts of its roots have been widely used in the treatment of inflammation, cancer, infectious diseases, hypercholesterolemia and hypertension [13,14]. The roots of this plant contain a plethora of bioactives, for instance different types of flavonoids such as baicalein and wogonin [15]. Recently, we showed that several flavonoids including quercetin, fisetin [16,17] and baicalein [18] possessed significant antiviral activities against dengue viruses (DENV).

Dengue is a serious viral disease in the tropical and subtropical regions of the world, and accounts for ~500,000 hospital admissions annually and consumes massive hospital resources [19]. Dengue is currently one of the most rapidly spreading mosquito-borne diseases that is estimated to pose health threat to ~2.5 billion people living in endemic regions [19,20]. Dengue virus (DENV) is an enveloped virus belonging to the *Flaviviridae* family with four distinct serotypes (DENV-1, DENV-2, DENV-3 and DENV-4) [19]. Currently, there is no specific anti-dengue medication available and treatment is only supportive [21]. In addition, there is no approved dengue vaccine available heretofore. Hence, there is an urgent and immediate necessity to explore an effective antiviral strategy against dengue. Since, single molecule antiviral drug development is a cumbersome, time-consuming and highly expensive endeavour, an efficient traditional herbal preparation could be an immediate alternative. Herein, we investigated the potentials of aqueous extract of *S. baicalensis* against the four DENV serotypes in regards to inhibition of different stages of replication *in vitro*.

## Methods

### Plant extract

*S. baicalensis* dried ground roots extract (AHPE-XA-09) used in the current investigation was provided by Herbitec Sdn Bhd (Malaysia), prepared using its own propriety method. Briefly, microwave energy generated steam was used to prepare the aqueous extract. The extract prepared in deionized water as a stock solution with concentration 60 mg/ml, was stored at −20°C. Before use in the experiments, the stock solution was clarified and sterilized using a syringe filter with 0.2 micron pore size (Millipore, MA, USA). Eagle’s minimum essential medium (EMEM) (Gibco, NY, USA) was used as a diluent to prepare the different concentrations of the extract.

### Standardization of extract and mass spectrometry

Identification and quantitation of the active compound, baicalein, in AHPE-XA-09 was performed by liquid chromatography-mass spectrometry-mass spectrometry (LC/MS/MS). Briefly, the concentration of pure baicalein ranging from 0.5 μg/mL to 20 μg/mL (Indofine Chemical Company Inc., New Jersey, USA) was dissolved in methanol (BDH Laboratory Supplies, England) and injected (10 μl) into the Applied Biosystems 3200 LC/MS/MS System (California, USA). Compound separation was achieved on a Gemini C6-Phenyl 110A column (150 mm×2.00 mm, particle diameter of 5 μm) (Phenomenex, USA) with a mobile phase composed of 0.1% formic acid (BDH Laboratory Supplies, England): acetonitrile (BDH Laboratory Supplies, England) at a flow rate of 0.50 mL/min. The transition ions and retention times were used as a detection point for baicalein peak. The transition ions were monitored in the multiple reaction monitoring modes (MRM). The area under the curve based on the different intensity height in the chromatogram of the different concentrations of baicalein was used to plot a standard linear regression curve. The AHPE-XA-09 (100 μg/mL lyophilized powder) was dissolved in methanol, filtrated through 0.45 μm PVDF filter (Whatman, USA) and was subsequently injected into the LC/MS/MS System. Inverse prediction method for the best fit linear regression curve generated from the pure baicalein was used for quantitation of the concentration of baicalein in AHPE-XA-09.

### Cells and virus

C6/36 mosquito cell line was used for the propagation of all DENV isolates used in the investigation [16]. Vero (African green monkey kidney) cell line was used for the evaluation of antiviral activity as previously described [18]. The cell lines were maintained and propagated in EMEM (Gibco, NY, USA) containing 10% fetal bovine serum (FBS, Gibco, NY, USA). The C6/36 and Vero cells were incubated at 28°C and 37°C respectively, in the presence of 5% CO_2_. At the time of virus inoculation and antiviral assays, the concentration of FBS was reduced to 2%. We used four different clinical DENV isolates representing the four serotypes of DENV (DENV-1, DENV-2, DENV-3 and DENV-4) in current investigation which identified by our group using full genome sequencing method. All the four clinical isolates propagated and maintained as previously described [17]. After titration of the virus isolates, the stocks were stored at −70°C until further use in the experiments.

### *In vitro* cytotoxicity assay

The cytotoxic potential of AHPE-XA-09 extract against Vero cells was determined using the MTT assay as described previously [19]. Briefly, the confluent Vero cells in 96-well microplates were treated with different concentrations of AHPE-XA-09 in triplicates. The treated cells were incubated with the extract for four days, which was similar to the time period for the antiviral assay at 37°C followed by the addition of 15 μl of MTT solution to each well. The microplate was further incubated at 37°C for four hours and subsequently a solublization/stop solution was added. The optical density (OD) of all the wells including non-treated cells was read using a plate reader (TECAN, Mannendorf, Switzerland) at 570 nm. The cytotoxicity of AHPE-XA-09 was calculated using Graph Pad Prism 5 (Graph Pad Software Inc., San Diego, CA).

### Antiviral activity assays

The effect of AHPE-XA-09 on intracellular replication of DENV was examined in Vero cells plated on 24-well cell culture microplate. After attaining 80% confluency, virus inoculum consisting of 200 FFU of each DENV serotype was added to each well. Viruses were allowed to adsorb to the cells for 1 hour at 37°C. Unadsorbed viruses were removed by rinsing the cells with sterile PBS twice. Different concentrations of AHPE-XA-09 were mixed with 1.5% carboxymethylcellulose (CMC) containing a cell-growth medium supplemented with 2% FBS and the plates were incubated at 37°C for four days. DENV foci were visualized as described previously [19]. The number of foci formed was expressed as foci-forming unit (FFU). The antiviral effects of AHPE-XA-09 was measured by calculating the percentage of foci reduction (% RF) against the controls maintained in parallel using the following formula [16]; RF (%) = (C-T) × 100/C, where, C is the mean of the number of foci from triplicates without AHPE-XA-09 added and T is the mean of the number of foci from triplicates of each treatment with AHPE-XA-09 extract.

### Time of addition studies

#### Prophylactic activity assay

The effects of prophylactic activity of AHPE-XA-09 prior to DENV infection were examined by treating a confluent monolayer of Vero cell line with different concentrations of AHPE-XA-09 for 5 h before DENV inoculation. The treatment medium was aspirated after 5 h, and the cells were washed twice with sterile PBS, and then infected with 200 FFU of each DENV serotypes. The microplate was kept at 37°C for 1 h to allow virus adsorption. Following virus adsorption, the infected monolayer was rinsed twice with sterile PBS and supplemented with 2% FBS containing EMEM with 1.5% CMC and the corresponding concentrations of the extract. Later, the plates were incubated at 37°C for 4 days in the presence of 5% CO_2_. Viral foci were stained and counted as described above.

#### Anti-adsorption activity assay

The effect of AHPE-XA-09 against DENVs adsorption and attachment to the Vero cells was determined by simultaneously adding the different concentrations of AHPE-XA-09 extract and 200 FFU DENV to Vero cells. The treated cells were washed with PBS after 1 h incubation at 37°C and supplemented with 2% FBS containing EMEM with 1.5% CMC. The plates were incubated at 37°C for 4 days with 5% CO_2_. Viral foci were stained and counted as described above.

### Extracellular virucidal activity

The potential direct virucidal effects of AHPE-XA-09 on DENV were evaluated by treating the viral suspension with 200 FFU of DENV with increasing concentrations of AHPE-XA-09 for 2 h at 37°C. The confluent Vero cells in 24-well plate were infected with the AHPE-XA-09-treated viral suspensions. After 1 h of adsorption at 37°C, the cells were washed twice with PBS and overlaid with 1.5% CMC-containing cell culture medium. The microplate was incubated for 4 days in a humidified 37°C incubator in the presence of 5% CO_2_. The viral foci were visualized as described above.

### Quantitative real-time polymerase chain reaction (qRT-PCR)

AHPE-XA-09 effects on DENV RNA production was determined using qRT-PCR as previously described [22,23]. Different primer sets for detection of the 4 different DENV serotypes were used based on previous designed primers [22,23] with some minor modifications (Table 1). This experiment was undertaken to confirm the foci forming assay results.

**Table 1:** Primer sequences used for q-RT-PCR of DENV

**Virus serotype**	**Primer sequence**
DENV-1	Forward: 5′ CAA TAT GCT GAA ACG CGC GAG AAA 3′
	Reverse: 5′ GCT CCA TTC TTC TTG AAT GA 3′
DENV-2	Forward: 5′ CAA TAT GCT GAA ACG CGA GAG AAA 3′
	Reverse: 5′ AAG ACA TTG ATG GCT TTT GA 3′
DENV-3	Forward: 5′ CAA TAT GCT GAA ACG CGT GAG AAA 3′
	Reverse: 5′ GAA GGT TCC CCA TCT AGC CA 3′
DENV-4	Forward: 5′ GAA GTG AAA ACA TGT CTG TGG CCC A 3′
	Reverse: 5′ TTC ACA GCA CAA TTA CCG CCA G 3′

### Statistical analysis

The half maximal cytotoxicity concentration (CC_50_) and half maximal inhibitory concentration (IC_50_) were used as the main parameters in this investigation. Selectivity index value (SI) was determined as the ratio of CC_50_/IC_50_ of the plant extract. GraphPad PRISM for Windows, version 5 (GraphPad Software Inc., San Diego, CA, 2005) was used for all statistical analyses.

## Results and Discussion

### Cytotoxicity of *S .Baicalensis* on Vero cells

**Figure 1:**
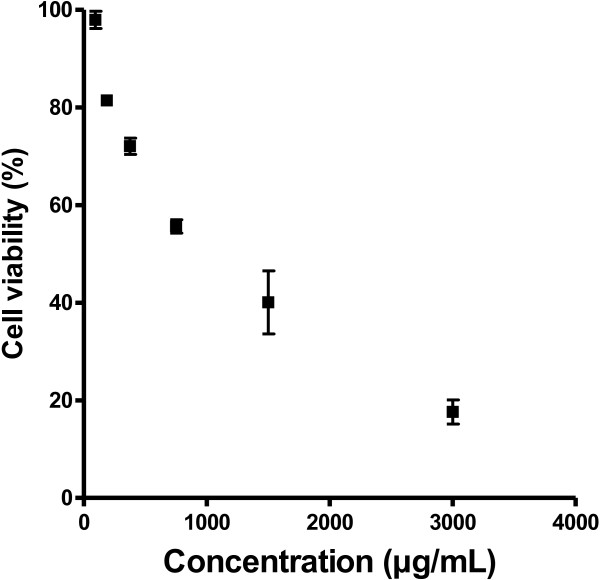
**Cytotoxicity effects of *S. baicalensis *extract, AHPE-XA-09, on Vero cells.** Data are presented as percentage of cell viability from triplicate assay.

The toxicity of AHPE-XA-09 extract was measured against Vero cells using the MTT assay. We found that 72 ± 2.9% of the cells were viable after 4 days of treatment with AHPE-XA-09 at concentration 375 μg/mL. Also, we found that in presence of 187.5 μg/mL the viability of treated cells was 81.4 ± 1.3. The half maximal cytotoxic concentration (CC_50_) value for AHPE-XA-09 was at 912.6 μg/mL (Figure 1). This suggests that AHPE-XA-09 at concentrations <375 μg/mL is in general non-cytotoxic and could be well-tolerated by Vero cells.

### Antiviral assays and mechanisms of action of AHPE-XA-09 against DENV

The foci-forming unit reduction assay (FFURA) together with qRT-PCR, were used to evaluate the *in vitro* anti-dengue activity of the aqueous extract of *S.baicalensis*. The extract was evaluated for its i) protective prophylactic effect ii) activity against virus adsorption to cells, iii) ability to inhibit replication after virus adsorption to the cells and iv) direct virucidal effect. The *S. baicalensis* extract, AHPE-XA-09, exhibited a dose-dependent inhibition effect against all the four DENV serotypes in Vero cells with IC_50_ values as presented in Table 2. It was demonstrated that the plant extract showed antiviral activity against the different DENV serotypes used at all stages of *in vitro* replication, although the most significant effects were observed when the extract was evaluated during the time of virus adsorption (Table 2).

**Table 2:** **IC**_**50 **_**values of the *S. baicalensis *extract, AHPE-XA-09, against the different stages of replication of DENV serotypes**

**Antiviral activity**	**Prophylactic activity IC**_**50 **_**(μg/mL)**	**Anti-adsorption activity IC**_**50 **_**(μg/mL)**	**After adsorption activity IC**_**50 **_**(μg/mL)**	**Direct virucidal activity IC**_**50 **_**(μg/mL)**
**Virus serotype**				
DENV-1	269.9	69.14	86.59	91.93
DENV-2	369.8	56.02	93.66	95.83
DENV-3	330.3	77.41	89.39	93.68
DENV-4	345.8	73.59	95.19	74.33

### Protective prophylactic effect

**Figure 2:**
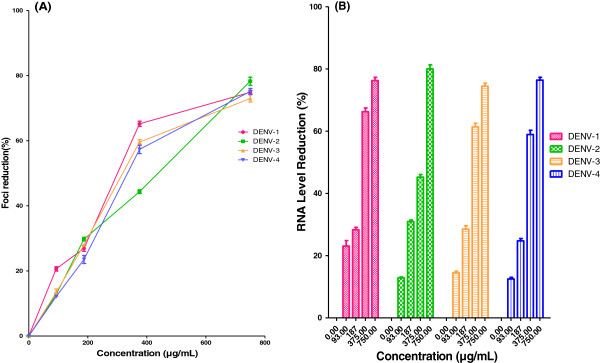
**Prophylactic effects of *S.baicalensis *extract against DENVs.** Foci forming unit reduction assay (FFURA) was performed to evaluate prophylactic activity of the plant extract on DENVs *in vitro* replication (**A**) and the respective DENVs RNA production levels were quantified by qRT-PCR for all four DENV serotypes (**B**). Data from triplicate assays were plotted using Graph Pad Prism Version 5 (Graph Pad Software Inc., San Diego, CA).

AHPE-XA-09 exhibited prophylactic effect against DENV replication in Vero cells, with the IC_50_ values ranging from 269.9 to 369.8 μg/mL for all the four DENV serotypes tested (Table 2, Figure 2A). The SI values for all the four DENV serotypes ranged between 2.47 and 3.38 (DENV-1, SI= 3.4; DENV-2, SI= 2.5; DENV-3, SI= 2.8; DENV-4, SI= 2.6). When the cells when pre-treated with the extract at a concentration of 750 μg/mL, the RNA levels in all the DENV serotypes decreased by >70% as compared to untreated cells (Figure 2B). These observations could be due to the uptake of the plant extract by the cells during pre-treatment and their potential activity against the intracellular replication of DENVs and/or could be related to the masking of the viral receptors on the cell surface due to competitive binding of the bioactives present in the plant extract. Nonetheless, the anti-DENV activity of AHPE-XA-09 via prophylactic treatment in Vero cells was relatively less significant as compared to the other treatments (Table 2). Therefore, it could be further improved probably by optimizing the time of exposure and dose for effective pre-treatment.

### Activity against virus adsorption to cells

**Figure 3:**
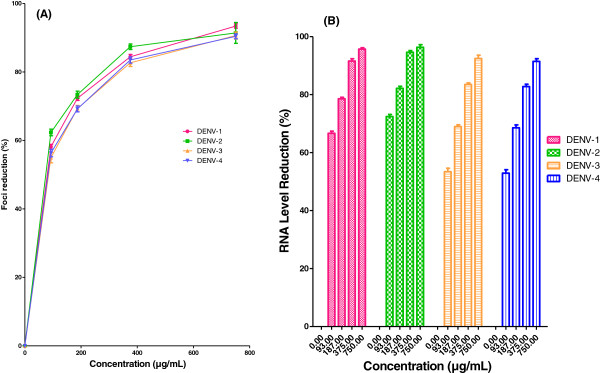
**Anti-adsorption activity of *S.baicalensis *extract against DENVs.** Foci forming unit reduction assay (FFURA) was performed to evaluate the effect of plant extract on DENVs adsorption and attachment to the Vero cells (**A**) and the respective DENVs RNA production levels were quantified by qRT-PCR for all 4 DENV serotypes (**B**). Data from triplicate assays were plotted using Graph Pad Prism Version 5 (Graph Pad Software Inc., San Diego, CA).

The antiviral activity of AHPE-XA-09 against DENV was found to be the most effective as it acts by inhibiting the virus adsorption to cells. The IC_50_ values ranged from 56.02 to 77.41 μg/mL for all the four DENV serotypes studied (Table 2, Figure 3A) with SI values ranged from 11.7 to 16.6 (DENV-1, SI= 13.2; DENV-2, SI= 16.3; DENV-3, SI= 11.8 and DENV-4 SI= 12.4). Interestingly, we also found that the DENV viral RNA reduced by ~68% to 82% at 187 μg/mL of AHPE-XA-09 added during the viral adsorption period (Figure 3B). This could be associated with the direct virucidal activity of the plant extract (Table 2), which possibly could have decreased the number of infectious DENVs particles during the adsorption time and/or binding to the cognate cellular receptors for DENVs. However, interfering with the virus attachment to the cells via binding to cell surface receptors and/or virus ligand could be another explanation for the anti-adsorption activity of the extract, which warrants further investigations.

### Ability to inhibit intracellular viral replication

**Figure 4:**
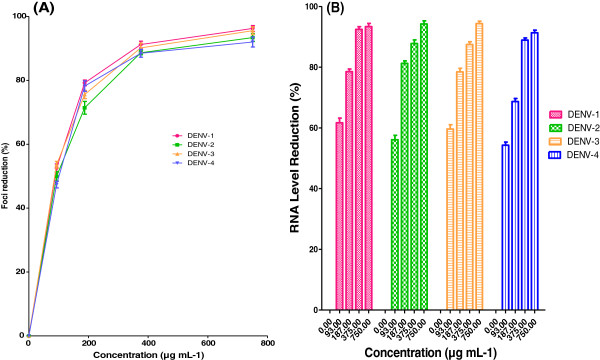
**Effects of *S.baicalensis *extract against DENVs intracellular replication.** Foci forming unit reduction assay (FFURA) was performed to determine the antiviral activity of the plant extract on DENVs after adsorption to the Vero cells (**A**) and the respective DENVs RNA production levels were quantified by qRT-PCR for all 4 DENV serotypes (**B**). Data from triplicate assays were plotted using Graph Pad Prism Version 5 (Graph Pad Software Inc., San Diego, CA).

Next, we set out to investigate the intracellular anti-dengue credentials of the plant extract DENV-infected Vero cells. Following the addition of AHPE-Xa-09 subsequent to DENV infection of Vero cells, we noticed an inhibition of DENV replication with IC_50_ values ranging from 86.59 to 95.19 μg/mL for the different DENV serotypes examined (Table 2, Figure 4A). The extract in this experiment showed SI values ranging from 9.5 to 10.5 (DENV-1, SI= 10.5; DENV-2, SI= 9.7; DENV-3, SI= 10.2 and DENV-4 SI= 9.6). The findings were convincingly supported by the results from qRT-PCR analysis, which showed a decrease of viral RNA yield by >50% at 93 μg/mL of the extract used, and a reduction of viral RNA by at least 90% at 750 μg/mL of the extract used as compared to the non-treated cells (Figure 4B). Similar findings have been reported by others where the extract from the roots of *S. baicalensis* have shown antiviral activity against respiratory syncytial virus (RSV) with a SI value of 10.7 [12]. It has also been established that a flavone from the roots of *S .baicalensis* possessed significant antiviral activity against influenza viruses when used after virus adsorption to the susceptible cell line [24].

### Direct virucidal effects

**Figure 5:**
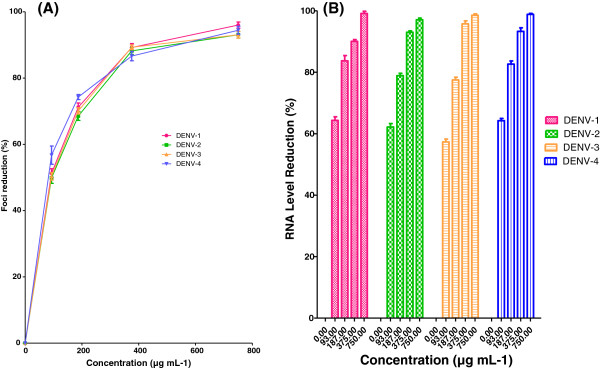
**Direct virucidal activity of *S. baicalensis *extract against DENVs.** Foci forming unit reduction assay (FFURA) was performed to evaluate the direct virucidal effects of the plant extract on DENVs (**A**) and the respective DENVs RNA production levels were quantified by qRT-PCR for all 4 DENV serotypes (**B**). Data from triplicate assays were plotted using Graph Pad Prism Version 5 (Graph Pad Software Inc., San Diego, CA).

It was demonstrated that AHPE-Xa-09 exhibited a potent extracellular anti-DENVs effect against all the four DENV serotypes. Inhibition of DENV replication in Vero cells was significant when AHPE-XA-09 was pre-incubated with the viruses, with IC_50_ values ranging from 74.33 to 95.83 μg/mL for the different DENV serotypes (Table 2, Figure 5A). The SI values of the extract in this part of the investigation ranged between 9.5 and 12.3 (DENV-1, SI= 9.9; DENV-2, SI= 9.5; DENV-3, SI= 9.7; DENV-4, SI= 12.3). The extract at 187 μg/mL decreased the RNA production of DENVs at the range of 77–83% (Figure 5B) as compared to the non-treated virus inoculum.

### Identification and quantitative analysis of baicalein in AHPE-XA-09

**Figure 6:**
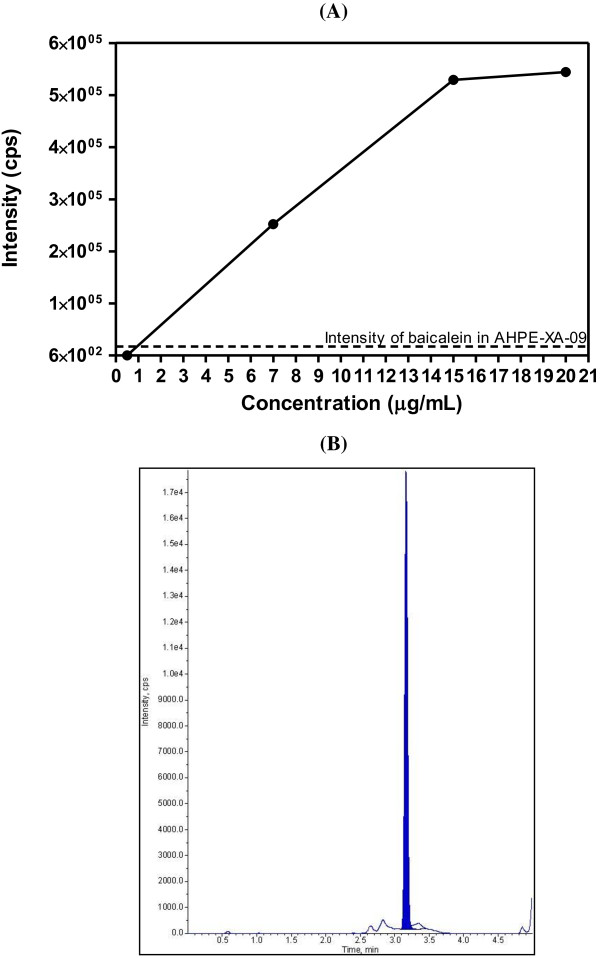
**Standard curve of the pure baicalein and LC/MS/MS chromatogram of AHPE-XA-09.** Pure baicalein standard linear regression curve was plotted from the area under the curve based on the different intensity height determined from the chromatogram of the different concentrations of pure baicalein. The intensity of baicalein in the AHPE-XA-09 was extrapolated from the plot (indicated by a dot line) (**A**). LC/MS/MS chromatogram shows the intensity and retention time of baicalein in the AHPE-XA-09 (**B**).

LC/MS/MS of the pure baicalein (0.5–20 μg/mL) was initially performed to establish a standard curve using area under the curve based on the different intensity height in the chromatogram of the different baicalein concentrations. A linear plot with R^2^ value of >0.996 was obtained. A transition ion with the MRM of mass-to-change-ratio (m/z) 270.9/122.9 atomic mass units (amu) and retention time of 3.14 minutes was monitored for pure baicalein. This enabled us to identify and confirm the presence of baicalein in AHPE-XA-09. Using the standard curve plotted from pure baicalein (Figure 6A), the amount of baicalein in 100 μg/mL of AHPE-XA-09, which showed intensity of 1.80 × 10^4^ counts per second (cps), was at 1.03 μg/mL (Figures 6A and 6B). The amount of baicalein present in AHPE-XA-09 was consistent with those reported from extraction of *S. baicalensis* roots using many other methods [25,26].

Recent lines of evidence suggest that certain compounds from the *S. baicalensis* extract, such as baicalein or chrysin, showed antiviral activity against hepatitis A virus (HAV), which is an RNA virus. However, the mechanism(s) underlying the mode of action of these compounds remain elusive [27]. Indeed, antiviral activity of 5,7,4'-trihydroxy-8-methoxyflavone a flavonoid from the roots of *S. baicalensis* was shown against influenza viruses [24,28].

Recently, we have shown the novel antiviral activity of pure commercial baicalein against DENV-2 (NGC strain) [18]. Therefore, our current investigations point to the potential use of cold water extract of the roots of *S. baicalensis*, AHPE-XA-09 as one of the main source for baicalein, although some other bioactives present in the extract could also possess anti-DENV activities. The anti-DENV activities of S*. baicalensis* extract were noticeable at all stages of *in vitro* infection; when the cells were treated after virus adsorption, during the virus adsorption or even prior to virus infection. The tested extract exerted significant direct virucidal effects on extracellular free DENVs particles, which is a notable ability for anti-dengue candidate to neutralize free viruses at viremic stages. The mechanism of how the extract precisely inhibits DENVs replication remains ambiguous. However, our findings suggest that one possible mechanism whereby the *S. baicalensis* extract acts against DENV replication could be attributed to its ability to bind and/or to inactivate certain important structural and/or non-structural protein(s) of DENVs. Such inhibitory mechanism has previously been reported with some flavonoids such as pinostrobin against DENV NS2B/NS3 protease [29]. Moreover, it has been demonstrated that baicalein, as a main flavonoid found in the tested extract, is able to bind to HIV-1 integrase [30]. On the other hand, the activity of flavonoids such as wogonin and baicalein has been demonstrated against cellular DNA and RNA besides their ability to inactivate the cellular RNA polymerases [31-33]. Therefore, it may be another potential mechanism for the tested plant extract and its constituents to inhibit the DENV replication via interference with DENV RNA polymerase and/or viral RNA, which is open for future investigations.

## Conclusions

In conclusion, the cold aqueous extract of the roots of *S. baicalensis*, one of the traditional Chinese medicinal herbs used to treat infectious diseases, exhibited a novel anti-dengue activity against all four serotypes of DENV *in vitro*. Our investigations have shown that this extract specifically targeted different stages of DENV infection and replication *in vitro*. Our study also demonstrated the potent direct virucidal activity of *S. baicalensis* extract, which serves as an important criteria for anti-dengue drug development as it could neutralize extracellular DENVs circulating in viremic patients. Our findings also highlight the potentials of *S. baicalensis* aqueous extract for use as anti-dengue agent, and the presence of baicalein, a known flavonoid with anti-dengue virus replication properties perhaps is one of the possible naturally active antiviral constituents.

## Competing interests

SAB acted as consultant to HSB for the study.

## Authors’ contributions

KZ designed and carried out the antiviral studies and drafted the manuscript. BTT carried out the virus propagation and antiviral studies. SSS, MBD, TKK participated in the quantitative RT-PCR and statistical analyses and edited the manuscript. THL and NAR participated in plant extraction. MHS carried out the extract HPLC analysis. SAB conceived the whole study and edited the manuscript. All authors read and approved the final manuscript.

## Pre-publication history

The pre-publication history for this paper can be accessed here:

http://www.biomedcentral.com/1472-6882/13/91/prepub
